# The Hidden Brain: Uncovering Previously Overlooked Brain Regions by Employing Novel Preclinical Unbiased Network Approaches

**DOI:** 10.3389/fnsys.2021.595507

**Published:** 2021-04-21

**Authors:** Sierra Simpson, Yueyi Chen, Emma Wellmeyer, Lauren C. Smith, Brianna Aragon Montes, Olivier George, Adam Kimbrough

**Affiliations:** ^1^Department of Psychiatry, School of Medicine, University of California, San Diego, San Diego, CA, United States; ^2^Department of Basic Medical Sciences, College of Veterinary Medicine, Purdue University, West Lafayette, IN, United States; ^3^Weldon School of Biomedical Engineering, Purdue University, West Lafayette, IN, United States; ^4^Purdue Institute for Inflammation, Immunology, and Infectious Disease, West Lafayette, IN, United States

**Keywords:** iDISCO, fMRI, immunohistochemistry, modularity, graph theory, network neuroscience, iDISCO+

## Abstract

A large focus of modern neuroscience has revolved around preselected brain regions of interest based on prior studies. While there are reasons to focus on brain regions implicated in prior work, the result has been a biased assessment of brain function. Thus, many brain regions that may prove crucial in a wide range of neurobiological problems, including neurodegenerative diseases and neuropsychiatric disorders, have been neglected. Advances in neuroimaging and computational neuroscience have made it possible to make unbiased assessments of whole-brain function and identify previously overlooked regions of the brain. This review will discuss the tools that have been developed to advance neuroscience and network-based computational approaches used to further analyze the interconnectivity of the brain. Furthermore, it will survey examples of neural network approaches that assess connectivity in clinical (i.e., human) and preclinical (i.e., animal model) studies and discuss how preclinical studies of neurodegenerative diseases and neuropsychiatric disorders can greatly benefit from the unbiased nature of whole-brain imaging and network neuroscience.

## Introduction

**FIGURE 1:**
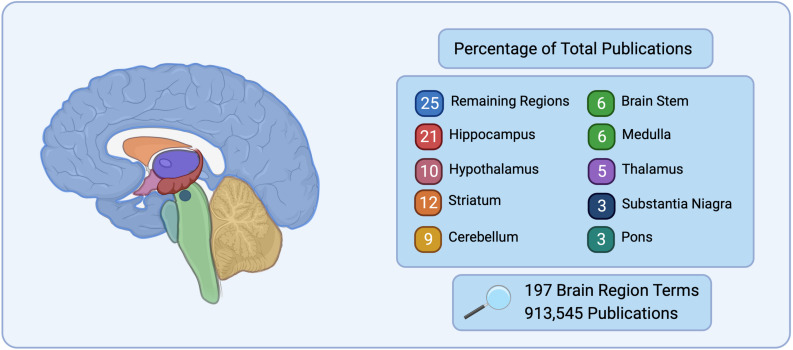
Number of publications listed on PubMed for 197 brain region terms searched for based on the Allen Mouse Brain Atlas (Allen Institute for Brain Science, 2004) nomenclature. The top nine brain regions represent 75% of the total publications, while the remaining 188 regions searched only represent 25% of total publications.

Historically, neuroscience has focused on specific regions of the brain such as the hippocampus for learning and memory (Scoville and Milner, 1957; Milner et al., 1998; Bird and Burgess, 2008), the hypothalamus for basal survival functions and motivated behavior (Swanson, 2000; Sternson, 2013), and the cerebellum for sensorimotor control (Buckner, 2013). These regions have distinct morphology and are large in comparison to the rest of the brain, making them simple targets for early exploration in neuroanatomy, staining, and electrophysiology techniques. This approach has left many regions understudied. This is highlighted in Figure 1, where during a search of 197 brain

regions, 75% of the publications in PubMed were found to correspond to only 9 brain regions, while the remaining 188 brain regions belonged to 25% of the search results. It is the natural progression of good science to continue the examination of brain regions or circuits that have prior evidence to suggest their importance to a given disease, however this neglects a vast array of brain regions and circuits that may also be critical.

Until recent years there was a lack of the technology and computing power necessary to assess the whole brain in a truly unbiased manner, as well as to interrogate the interconnectivity and functionality of the entire neural network. Probing the brain as an integrated organ allows for novel methods to map, record, and analyze interactions between and within regions. The vastness and complexity of large data sets from the brain have required new ways to reduce dimensionality without significant data loss or bias (Hinton and Salakhutdinov, 2006; Cunningham and Yu, 2014; Mwangi et al., 2014; Beyeler et al., 2019; Crimi et al., 2019). Recent advances in computational tools have enabled the processing of dense information clouds following modulation of circuits, as well as the ability to create comprehensive connectivity maps to make network analysis possible (Thompson et al., 2007; Hawrylycz et al., 2012; Wohnoutka et al., 2014; Bakken et al., 2016; Bassett and Sporns, 2017).

Preclinical (i.e., animal model) whole-brain imaging approaches are now capable of providing immense data sets of neural activity with improved resolution (either timescale or brain structure resolution) than previously available. Additionally, advances in computational analysis of brain-wide function offer unique ways to assess brain activity from these data sets, during critical neural states, in an unbiased manner. Together, these approaches can help identify previously overlooked brain regions that may be critical for given disease states and help to contextualize the contribution of heavily studied brain regions to overall brain function. This review will cover the use of preclinical whole brain imaging and neural network tools to assess neural activity of the brain.

### Circuit Manipulation and Whole Brain Imaging Methods in Preclinical Neuroscience

The ability to alter the excitatory or inhibitory properties of a neuron is critical to the study of the brain. Multiple circuit and imaging approaches can be leveraged to measure and manipulate brain activity. An early example of these tools is electrodes, which are a robust way to electrically stimulate or inhibit cells. Deep-brain stimulation using implantable electrodes has been highly effective in the treatment of epilepsy (Boon et al., 2009; Zangiabadi et al., 2019) but this technique requires invasive surgery and implantation. Clinically, transcranial magnetic stimulation (TMS) and focused ultrasound (FUS) are alternative ways to stimulate the brain without the need for invasive implantation (Lynn et al., 1942; Fry et al., 1955; Fishman and Frenkel, 2017). Further discussion of the methods and the progression of emergent technologies to manipulate the brain will be discussed in the following section.

Preclinical methods to influence neural signaling include electrophysiology, pharmacology, and more recently, optogenetics and chemogenetics for more specific neural stimulation. Optogenetics, leverages light sensitive ion channels to alter the activity of brain regions with a high degree of specificity. Light-gated cation channels such as excitatory channelrhodopsin-2 (ChR2) and inhibitory channels (i.e., halorhodopsin and archaerhopsin) can easily be integrated into neural tissue using viral vectors to confer temporal and spatial specificity (Boyden et al., 2005; Deisseroth et al., 2006; Zhang et al., 2006; Gradinaru et al., 2008; Miesenbock, 2009; Chow et al., 2010; Abe et al., 2012). Optogenetics has been employed to map neural circuitry, identify behaviors associated with poorly understood brain regions, and develop animal models to better understand of the contributions of a specific brain region or cell type in behavioral and emotional states (Bernstein and Boyden, 2011; Tye et al., 2011, 2013; Kim et al., 2013; Ye et al., 2016).

Furthermore, the combination of optogenetics and fMRI (ofMRI) has enabled preclinical investigation of the functional connectivity between neural circuits with spatial and temporal specificity (Lee et al., 2010; Abe et al., 2012; Lee, 2012; Lin et al., 2016; Lein et al., 2018). The use of ofMRI enables millisecond-timescale modulation of activity in the intact brain and has elucidated novel global and local fMRI signals driven by populations of optogenetically defined neurons. Development of this paradigm has improved the understanding of widespread brain responses to specific local activation (Weitz and Lee, 2013).

Chemogenetics, like optogenetics, has been used to identify distinct neural circuits associated with behavioral and emotional states (Strader et al., 1991; Bishop et al., 1998; Chen et al., 2005; Sternson and Roth, 2014; Zhu et al., 2014; Vetere et al., 2017). A benefit of chemogenetics is that the receptors can be genetically encoded and do not require the implantation of a light delivery device; however, the timescale is different from the millisecond activation of optogenetics, ranging 1–6 h for CNO DREADDS, and 5–60 min for KORD-activated DREADDS (Alexander et al., 2009; Guettier et al., 2009; Sternson and Roth, 2014; Roth, 2016; Urban et al., 2016; Whissell et al., 2016). Chemogenetics approaches can reduce surgical manipulation, leaving the brain tissue intact for later analysis, and simplify the associated behavioral assays. Additionally, these receptors can have a diverse range of cellular functions and signaling processes ranging from engineered kinases (Strader et al., 1991; Bishop et al., 1998; Chen et al., 2005), and G-protein coupled receptors (GPCRs) (Redfern et al., 1999; Armbruster et al., 2007; Alexander et al., 2009; Vardy et al., 2015) to ligand-gated ion channels (Lerchner et al., 2007; Arenkiel et al., 2008; Magnus et al., 2011) and the most commonly implemented DREADDs (Armbruster et al., 2007).

While optogenetics and chemogenetics focus on altering the excitability of the brain, visualizing the innate activation of neurons is just as crucial. Previously, the innate electrical activity of the brain was monitored by single electrodes or multielectrode arrays (Nicolelis and Ribeiro, 2002); however, this technique is limited by spatial specificity and is difficult to scale at the cellular level (Lewis et al., 2015). *Ex vivo* approaches, such as measurement of brain-wide protein from the immediate early gene *c-fos*, accomplish a snapshot of activity at a particular brain state (Ragan et al., 2012; Osten and Margrie, 2013) with intricate pipelines to employ serial sectioning and realignment (Mesina et al., 2016). However, this approach is limited to a generalized timescale with no way to repeatedly sample the same brain. The invention of two-photon calcium imaging allowed for some of the first *in vivo* visualizations of the activity of distinct neurons in brain tissue (Tsien, 1988). This real-time analysis revealed activity at the cellular and subcellular level. Calcium imaging can also be employed in *in vitro* studies in brain slices and *in vivo* preparations using two-photon microscopy (Denk et al., 1990; Mao et al., 2001; Oh et al., 2005; Benninger and Piston, 2013) or in combination with multielectrode recordings in freely moving animals to interrogate and reconstruct functional connectivity in real-time (Ozbay et al., 2018; Bonifazi and Massobrio, 2019).

Resting-state fMRI (R-fMRI) is another useful technique in comparing functional similarities across species. Xu et al. explored R-fMRI data from macaques and humans combined with a computational approach called joint embedding. They were able to assign common brain architecture features between human and macaque brains. Xu et al. (2020) further developed a Functional Connectivity Homology Index (FCHI) to quantify the cross-species similarities, pushing the limits of network analysis both within a species and between species. Additional methods to elucidate common features within fMRI data such as independent component analysis (ICA) can be employed as an exploratory method to reveal network patterning even when the stimuli are complex or are not time-locked to a specific event (Calhoun et al., 2001; Mckeown et al., 2003; Beckmann et al., 2005; Calhoun and Adali, 2012). This type of exploratory approach is data-driven, eliminating bias of a specific brain region or treatment/event (Mckeown et al., 1998). A complementary tool to ICA is Sparse Dictionary Learning (SDL) which is capable of evaluating functional networks with significant spatial overlap. However, ICA performs better in networks without spatial overlap (Zhang et al., 2019). Tools such as these are useful in bridging the cross-species gap that often emerges comparing preclinical models and human.

Another emerging technique in preclinical network science is 4D functional ultrasound (4DFUS) imagining of whole-brain for preclinical applications (Rabut et al., 2019). This approach developed by Rabut et al. implements multiplate wave transmissions on matrix arrays at thousands of frames per second to allow for volumetric recordings of blood volume changes in the brain with high resolution in both space and time. Ultrafast imaging relies on coherent compounding of backscattered echoes. The use of Hadamard coefficients can increase resolution without compromising the frame rate (Tiran et al., 2015). 4DFUS complements electrophysiological and optical methods because while those approaches provide similar specificity in terms of resolution, they lack the ability to expand monitoring to a larger-scale network.

Functional readouts provide insight to activity in the awake, behaving brain, but lack cellular resolution. Recent preclinical developments in tissue-clearing methods allow for three-dimensional imaging of the intact brain. There are many approaches for clearing tissues which include CLARITY (cleared lipid-extracted acryl-hybridized rigid immunostaining) (Chung et al., 2013), DISCO (Three-dimensional imaging of solvent-cleared organs) (Erturk et al., 2011, 2012), iDISCO (immunolabeling-enabled DISCO) (Renier et al., 2014), SHIELD (stabilization under harsh conditions via intra molecular epoxide linkages to prevent degradation) (Park et al., 2018), FocusClear (Fu and Tang, 2010), SeeDB (see Deep Brain) (Ke et al., 2013), FRUIT (Fructose Urea in α-Thioglycerol) (Hou et al., 2015), and CUBIC (clear, unobstructed brain imaging cocktails and computational analysis) (Susaki et al., 2015; Susaki and Ueda, 2016; Murakami et al., 2018; Matsumoto et al., 2019). The CLARITY approach consists of a hydrogel-based method which uses covalent linkage to an acryl-based hydrogel for complete lipid removal with limited structural damage and protein loss (Chung et al., 2013). The DISCO approach uses tetrahydrofuran, a dehydrating and delipidating agent, instead of an alcohol (Erturk et al., 2011, 2012). Further iterating on this method, Renier et al. (2014, 2016) developed iDISCO and iDISCO+, which allows for whole-mount immunolabeling of whole cleared organs and wide-scale mapping of brain activity by analysis of immediate early genes. As an alternative to the use of hydrophobic solvent clearing techniques, tissues can be impregnated with high-osmotic aqueous solutions for a hydrophilic approach with beneficial refractive indices (Tainaka et al., 2018). These methods include SeeDB (Ke et al., 2013), FRUIT (Hou et al., 2015), FocusClear (Fu and Tang, 2010), and CUBIC (Susaki and Ueda, 2016; Tainaka et al., 2018). For an extensive comparison of clearing techniques and microscopy applications (see Richardson and Lichtman, 2015; Silvestri et al., 2016; Vigouroux et al., 2017; Muntifering et al., 2018).

From circuit manipulation to novel clearing techniques the development of tools to enable the observation and alteration of an intact brain with a spatial and temporal resolution has encouraged the exploration of brain regions that were previously impossible to assess. However, the data that is produced by these techniques is immense and difficult to directly interpret. Advanced computational network-based approaches, especially with regard to brain clearing and neural activity, such as ClearMap (Renier et al., 2016) are necessary for taking full advantage of data from cleared brains.

### Computational Analysis of Neural Networks

The combination of preclinical whole-brain imaging approaches with advances in computational analysis of brain-wide function serves as a unique way to assess brain activity during critical neural states in an unbiased manner. Methods of unbiased assessment including fMRI, calcium imaging, and immediate early gene imaging, may provide a pivotal way to identify brain regions that have been overlooked previously due to technical difficulty or lack of interest that are critical for a given behavior or disease state. These types of imaging data can be studied in greater detail using network-based approaches.

In neuroscience, network-based approaches offer unique ways to assess neural activity at a brain-wide scale that may be critical for identifying aspects of disease, such as, such as improvement of treatment methods and identification of biomarkers (Lydon-Staley and Bassett, 2018; Zhang et al., 2020). Network neuroscience can be used to assess the structural or functional connectivity of the brain. Structural networks measure the physical connectivity of brain regions (i.e., do two brain regions have physical connections for direct communication) by identifying fiber tracts and axonal connections, whereas functional networks examine the correlative connectivity of neural activity between regions (i.e., are two brain regions usually activated simultaneously in a given state, suggesting direct or indirect communication) in an unbiased manner (Bullmore and Sporns, 2009; Vertes et al., 2012; Bassett and Sporns, 2017).

Networks measuring functional connectivity, using graph theory, can be employed to identify specific features of neural networks in more detail. Graph theory can be applied to neural network data across multiple levels (e.g., whole brain, regions, circuits, neurons). This approach models the pairwise relations between nodes, through connected edges (Sporns, 2018). When modeling the brain, the nodes can be individual neurons or specific anatomical brain regions, allowing for the scale that is often lost using traditional recording techniques. Edges are defined as the functional connectivity between neurons or brain regions, as measured by correlation of neural activity.

**FIGURE 2:**
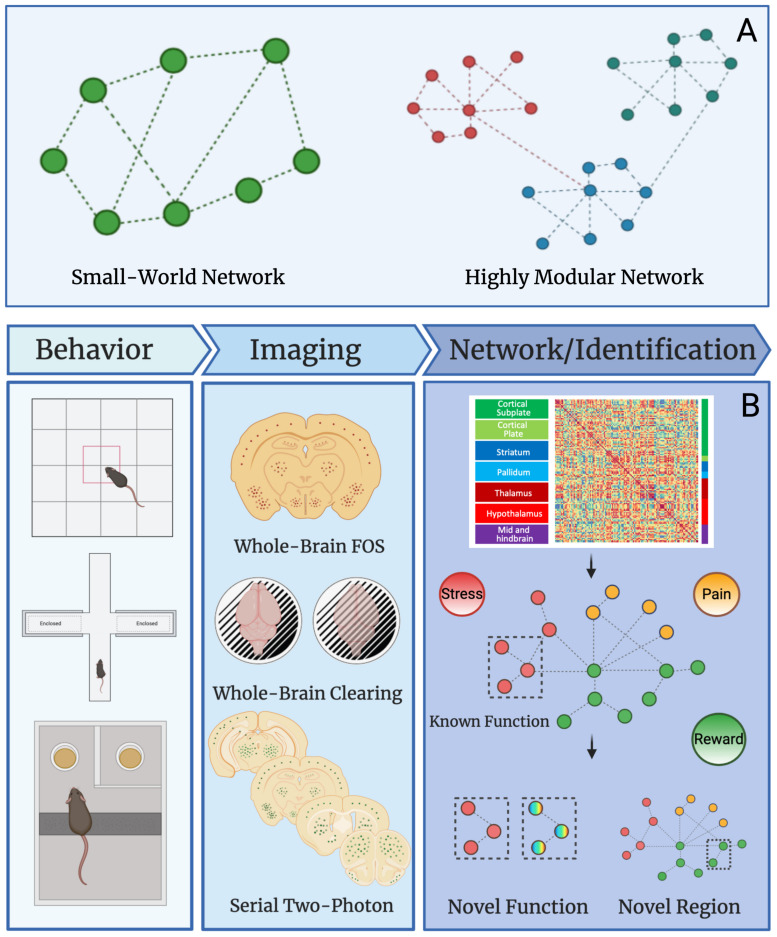
Network properties and uses in preclinical studies. **(A)** Graphical example of small-world and highly modular networks. **(B)** Workflow for preclinical network analysis using Fos as a marker for neural activity. Animals first undergo a behavioral, pharmacological, or alternative manipulation to induce neural activity. Brains are collected and then processed to identify Fos protein expression using one of several immunostaining and imaging strategies. The main strategies are: (1) traditional immunohistochemistry and microscopy on Fos stained sliced brain tissue, (2) whole brain immunostaining/clearing of Fos and light-sheet microscopy, and (3) serial two-photon imaging of fluorescent brain slices. Once data is collected from any given imaging strategy, functional connectivity networks can be delineated by calculating Pearson correlations of Fos activity from one brain region to another brain region across animals in a given treatment group. This is done for all brain regions expressing Fos to create a functional connectivity matrix. The functional connectivity matrix can be used to create a modular network that contains brain regions grouped based on function (e.g., stress, pain, or reward). Network analysis can then reveal novel functions for brain regions with other known roles and additionally, the function of overlooked and understudied brain regions can be identified. This workflow will be useful for identifying novel brain regions that contribute to neuropsychiatric diseases in the future.

Networks can be divided into modules (i.e., groups brain regions) of nodes (i.e., brain regions) that may share specific neural functions (Meunier et al., 2010). For instance, the nucleus accumbens and ventral tegmental area are both involved in reward processes and could be grouped together in a reward module as such for a given brain state. In human data, many neural networks show small-worldness and modular organization (Bassett and Bullmore, 2006; Sporns and Betzel, 2016). The “world” of a network is termed as “small” if the average number of connections between nodes (geodesic distance) is small relative to the total number of nodes within the network (Achard et al., 2006; Humphries and Gurney, 2008; Muldoon et al., 2016; Bassett and Bullmore, 2017). This means that most nodes are not connected to each other but are connected indirectly through the overall network through only a few connections. In contrast a highly modular network contains a large number of interconnected nodes and few intra-connected nodes (Stam, 2004; Ahn et al., 2010; Sporns and Betzel, 2016). A graphical representation of small-world networks and highly modular networks can be found in Figure 2A. To simplify these complex datasets, machine learning can be used. Machine and deep learning are among the novel computational methods employed to reduce network complexity. Although an in-depth discussion of these techniques is outside the scope of this review, we recommend Vu et al. (2018); Glaser et al. (2019), and Valliani et al. (2019) for further review of the topic.

Graph theory can be used illuminate other intrinsic qualities of the network, such as overall network efficiency (how easily information is exchanged between nodes) and node centrality (the most influential component of a node). In this context, the efficiency of a given network is characterized by the average of the shortest path lengths between any set of nodes (Sporns et al., 2004; Bassett and Bullmore, 2006). Node centrality is the output of the relative importance of a node for a given network. Centrality looks at several measurements such as degree, efficiency (Achard and Bullmore, 2007; Joyce et al., 2010), closeness, and betweenness of each node (Wang et al., 2010). Nodes with a high number of connections (lower path length) and that are central to the network (most important) are considered hubs. Groupings of important hubs with many connects are grouped together in something called a “rich club” to identify high-importance nodes (Guimera et al., 2005; Sporns et al., 2007; Vertes et al., 2014).

A network hub is a node with high intramodule connectivity (a provincial hub), high intermodule connectivity (a connector hub), or both high intra- and inter- module connectivity (a dual hub) (Guimera and Nunes Amaral, 2005; Guimera et al., 2005; Joyce et al., 2010; Stevens et al., 2012; Pedersen et al., 2020). In the context of the brain, hub brain regions represent the highest level of connectivity and are thought to be critical to the function of the neural network (Sporns et al., 2007; Rubinov and Sporns, 2010; Wheeler et al., 2013; Vetere et al., 2017; Kimbrough et al., 2020; Pedersen et al., 2020). Importantly, hubs identified to be crucial in neural networks have been demonstrated to be conserved across species and scales (Arnatkeviciute et al., 2019), both validating the use of the model and suggesting the importance of assessment of networks in disease states across species. To determine activity, immediate early genes have been leveraged, of which Fos is most widely recognized as a marker of activity. The nuclear staining of Fos also makes it an easy marker for imaging and computational analysis.

A key feature of imaging approaches paired with network analysis, whether in clinical or preclinical studies is the unbiased nature of the brain-wide activity being assessed, which can uncover important connectivity and function of the brain without relying on *a priori* selection of a specific circuit or region to study. Clinical and preclinical studies focused on neuropsychiatric disorders and other brain diseases can greatly benefit from taking advantage of network neuroscience to determine important connections between brain regions and potential hubs associated with a given brain state. Identification of critical hub brain regions within a given disease state help to delineate novel signaling pathways and find potential brain regions and/or therapeutic treatments that were previously overlooked.

### Applications of Network Neuroscience in the Treatment and Study of Disease

The primary application of network neuroscience over the last 10 years has been in human studies. One of the main ways to study functional connectivity of the human brain has been through R-fMRI, which can assess differences in default mode network (DMN) function among groups (Gonen et al., 2020). R-fMRI is not without issues, however, as there is a need to standardize the methods among research groups (Dinis Fernandes et al., 2020) and to determine the importance of time-varying functional connectivity measured at rest (Lurie et al., 2020). Using fMRI and DTI scans Akiki et al. (2018) described DMN abnormalities in PTSD patients through a novel network restricted topology approach. They combined fMRI, DTI, and graph theory to systematically examine DMN connectivity and its relationship with PTSD symptom severity. DMN abnormalities were observed in patients with severe PTSD and computational analysis revealed decreased overall interconnections within this group. Other neuropsychiatric disorders such as depression (Jacob et al., 2020), schizophrenia (Whitfield-Gabrieli et al., 2018) and anxiety (Qiao et al., 2017) have been studied similarly. Clinical approaches using machine and deep learning are applied to data collection in prior studies to identify novel markers and disease states. Jo et al. compiled functional scans of brains from Alzheimer’s disease patients and were able to develop a model to predict Alzheimer’s disease progression with a high level of confidence (Jo et al., 2019). Machine learning has also been applied to comorbid psychiatric disease that are difficult to tease apart such as anxiety and depression. This analysis clarifies distinct behavioral measures that contribute to the prediction and crucial mechanisms in one condition vs. the other (Richter et al., 2020).

Network neuroscience has been recently been applied to study preclinical animal models of disease. In preclinical research brain-wide neural networks can be assessed by pairing brain imaging techniques such as fMRI, traditional immunohistochemistry, single-cell whole-brain imaging, and two-photon imaging with network analysis. Using fMRI for preclinical network analysis, Gass et al. interrogated the difference in neural network reorganization between stress-resilient rats and stress-sensitive rats (Gass et al., 2016). This study identified alterations in the role of hubs in a default-mode-like-network sensitive vs. resilient rats that uncovered novel internodal shifts that would have been undetectable using traditional methods.

Calcium imaging is appropriate for the preclinical exploration of the brain; however, it is not applicable to clinical studies in humans. To overcome this issue, the most commonly used clinical neuroimaging techniques are MRI, fMRI, and DTI, which are all relatively fast and non-invasive (Kwong et al., 1992; Basser et al., 1994; Glover, 2011). Used in combination with traditional MRI, DTI extends the imaging capability of the whole brain *in vivo*. These approaches are beginning to bridge the gap between clinical and preclinical studies, enabling comparison of functional brain activity in human subjects as well as preclinical animal models (Denic et al., 2011; Glover, 2011).

Although preclinical calcium and fMRI imaging provide near instant time resolution, the resolution of individual brain regions is greatly reduced. Further, animals often need to be head fixed or anesthetized in order to record activity data, which greatly limits the ability to assess activity during complex behavioral tasks. Thus, there is value in taking advantage of postmortem immediate early gene protein signaling measurements of neural activity (e.g., tissue clearing techniques and two-photon imaging) to examine brain-wide neural activity.

In addition to the foundational studies focused on whole-brain imaging of Fos activity (Osten and Margrie, 2013; Renier et al., 2014, 2016), others have begun to combine Fos measures with functional connectivity and network analysis to assess activity in the brain. Traditional quantification of brain regions by staining for Fos after fear conditioning was used to establish a neural network associated with fear memory (Wheeler et al., 2013). Further assessment of the fear memory network using functional chemogenetic silencing of different network nodes *in vivo* aided in the identification of a novel causal role of the reuniens and laterodorsal thalamic nucleus in behavior for key hub brain regions predicted by network models (Vetere et al., 2017). Additionally, graph theory was applied to predict the influence of the hippocampus in driving transitions between non-dependent and dependent states leveraging a control theoretic approach (Brynildsen et al., 2020). Figure 2B represents the workflow from behavior through novel function and region detection. These studies provided a blueprint for using Fos immunostaining in network models that could be extended to the whole brain with advances in brain-wide imaging. There are some caveats to the use of Fos as a marker of activity. Not all immediate early genes are expressed similarly by all neurons (Gallo et al., 2018). Other immediate early genes such as Egr1 and Arc have been demonstrated to have distinct patterns on expression from Fos, and exhibit different timescales from the initiation of activity (Milbrandt, 1987). The present review has highlighted how network-based approaches in preclinical models of disease can examine brain-wide neural activity in an unbiased manner, which will help identify critical brain regions that may have been overlooked previously.

Recently, the combination of iDISCO+, light-sheet microscopy, functional connectivity, and graph theory has been used to reveal the neural network involved in alcohol abstinence. A massive increase in functional coactivation among brain regions and reduced modular structuring of the brain was found in the alcohol abstinence network (Kimbrough et al., 2020). Further, the study by Kimbrough et al. identified critical hub brain regions, validating regions known to play a role in alcohol withdrawal (e.g., the central amygdala; Gilpin et al., 2015) and identified some regions such as the parasubthalamic nucleus, tuberal nucleus, cortical amygdala, and intercalated amygdala that has been overlooked in alcohol studies and need more detailed research (Kimbrough et al., 2020). Combining single-cell whole-brain imaging approaches of immediate early gene immunostaining (e.g., iDISCO/light-sheet microscopy, traditional immunohistochemistry, or serial two-photon imaging) with network analysis can be used to identify novel brain regions of interest for various disease states and neuropsychiatric disorders that were previously overlooked, and also alternative functions for known regions that may contribute to disease progression (Figure 2).

As network neuroscience approaches develop, the way to analyze imaging data of disease states from the clinic has expanded. Neural networks provide unveil previously hidden contributors to disease states (Lydon-Staley and Bassett, 2018; Zhang et al., 2020). Neural network analysis of humans for various neuropsychiatric disorders such as depression, anxiety, post-traumatic stress disorder (PTSD), and Alzheimer’s disease have uncovered contributions of the brain to the disorders (Bashyal, 2005; Jo et al., 2019).

## Discussion

Past research in neuroscience has been heavily skewed to focus on a handful of major groups of brain regions and circuits (Figure 1). The use of single-cell whole-brain imaging combined with network analysis in preclinical models is a valuable tool moving forward in neuroscience research, especially when taking advantage of the synergy with other recently developed preclinical technologies. For example, pharmacological, chemogenetic, and optogenetics approaches can be combined with single-cell whole-brain imaging to examine how the modification of specific cell types or brain regions impact the connectivity and granularity of each hub within the whole brain. This approach provides a platform for testing brain regions identified by network analysis as potential hubs for a causal role in behavioral output. Additionally, the combination of single-cell whole-brain imaging and chemogenetics or optogenetics allows for modifying regions known to play a role in a given behavior to explore the interconnectedness of brain regions associated with the specific circuit but that is at a tertiary, quaternary, or further connection away from the region of interest. Functional read-outs such as calcium imaging can be used to elucidate neuron specificity at the cellular and local network levels within a given brain region in combination with the unbiased evaluation of brain-wide networks within the same animals.

There are some caveats to preclinical whole-brain imaging/network analysis approaches. First, depending on the method there will be limitations on the resolution of timescale (in the case of immediate early gene imaging) or brain region specificity (in the case of fMRI and calcium imaging). The issue of resolution suggests that there are benefits to both approaches and they can both prove informative in different aspects of the study of disease. In animal models, fMRI also restricts the type of behavior or brain state that can be examined due to the methods for imaging, whereas using immediate early genes requires postmortem tissue and does not allow for assessment of future behavior/brain activity. Another weakness with preclinical studies is the ability to translate the information from animal models to relevance in human disease states. However, development of methods for comparison of functional brain activity in human subjects with preclinical animal models has helped to alleviate these issues (Denic et al., 2011; Glover, 2011). Furthermore, important hubs identified using neural network methods are maintained across species and scales (Arnatkeviciute et al., 2019). Whole brain imaging and network analysis in preclinical animal models will only improve as time goes on and the issues are minimized.

Despite the limitations discussed above, network-based approaches in preclinical studies have the potential to make significant contributions to our understanding of how the brain functions during behavior and as a result of neuropsychiatric disease. In combination with other modern neuroscience techniques, whole-brain imaging and network analysis may vastly enhance our systems-based understanding of the brain in a way that was not previously available. Importantly, examining network function across the whole brain provides an unbiased examination of brain activity that will help to identify brain regions that are critical for function but have been previously overlooked.

## Author Contributions

SS and AK conceptualized the topic and wrote the manuscript. LS, YC, EW, and BA provided background research for the topic. SS designed the figures. SS, YC, EW, LS, OG, and AK edited the manuscript. All authors contributed to the article and approved the submitted version.

## Conflict of Interest

The authors declare that the research was conducted in the absence of any commercial or financial relationships that could be construed as a potential conflict of interest.
